# Spontaneous Rupture of Pancreatic Pseudocyst: Report of Two Cases

**DOI:** 10.1155/2016/7056567

**Published:** 2016-03-20

**Authors:** Ricardo Rocha, Rui Marinho, António Gomes, Marta Sousa, Nuno Pignatelli, Carla Carneiro, Vitor Nunes

**Affiliations:** Surgery Department, Cirurgia B, Hospital Prof. Dr. Fernando Fonseca, 2720-276 Amadora, Portugal

## Abstract

*Introduction*. Pancreatic pseudocysts are a common complication of acute pancreatitis. Pancreatic pseudocyst's natural history ranges between its spontaneous regression and the settlement of serious complications if untreated, such as splenic complications, hemorrhage, infection, biliary complications, portal hypertension, and rupture. The rupture of a pancreatic pseudocyst to the peritoneal cavity is a dangerous complication leading to severe peritonitis and septic conditions. It requires emergent surgical exploration that is often of great technical difficulty and with important morbidity and mortality.* Case Study*. We present two cases of spontaneous rupture of pancreatic pseudocysts, managed differently according to the local and systemic conditions.* Conclusion*. The best surgical choice is the internal drainage of the cyst to the GI tract; however, in some conditions, the external drainage is the only choice available.

## 1. Introduction

Pancreatic pseudocysts are a common clinical problem after acute pancreatitis, with an estimated prevalence of 6 to 18.5%. In chronic pancreatitis its prevalence is higher, ranging from 20 to 40% [1].

Pseudocyst of the pancreas is an encapsulated collection of fluid with a well-defined inflammatory wall usually outside the pancreas with minimal or no necrosis, usually occurring more than 4 weeks after onset on acute pancreatitis [2].

Pancreatic pseudocysts are caused by pancreatic ductal disruption following increased pancreatic ductal pressure, either due to stenosis, calculi, or protein plugs obstructing the main pancreatic ductal system or as a result of pancreatic necrosis following an attack of acute pancreatitis [3].

Its clinical presentation may range from a completely asymptomatic patient to the onset of serious complications.

About 8% to 70% of all pancreatic pseudocysts may have spontaneous regression [1]. The vast majority is now treated electively; however, the incidence of complications needing emergent surgical management is not despicable [3].

Spontaneous rupture of pancreatic pseudocysts is a serious complication leading to severe peritonitis and the need of emergent surgical exploration.

## 2. Case Reports

### 2.1. Case 1

**Figure 1:**
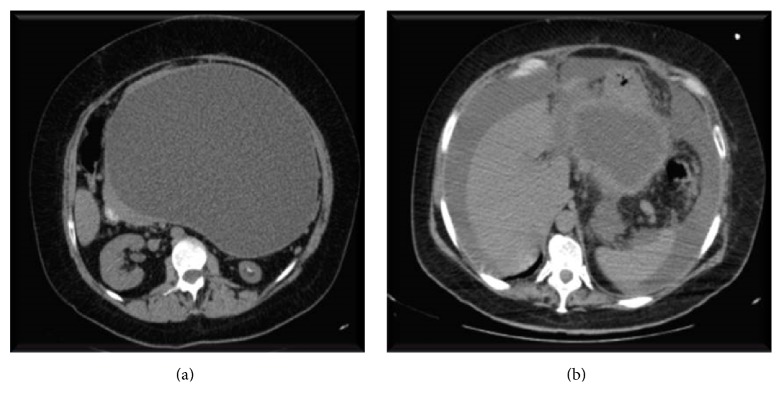
CT scan before and after pseudocyst rupture.

A 50-year-old female, with previous history of morbid obesity, biliary lithiasis, and an episode of mild acute biliary pancreatitis, treated 2 months previously, presented to the emergency room (ER) with diffuse abdominal pain. Physical examination revealed upper abdominal tenderness, without other findings. The CT scan revealed a large pancreatic pseudocyst, with 23 cm of major axis, with a wall greater than 5 mm (Figure 1(a)). She was admitted to the surgical department for elective drainage.

On the second day in the hospital, she became hypotensive and tachycardiac, with sudden installation of acute abdominal pain, without any history of trauma. CT scan revealed considerable amount of free fluid in the peritoneal cavity, with substantial reduction of pseudocyst dimension, compatible with the diagnosis of ruptured pseudocyst into the peritoneum (Figure 1(b)).

She was then submitted to an emergent laparotomy, where an intense chemical peritonitis was found, caused by leaking of pancreatic secretions, and there was a clearly identifiable orifice of drainage of the pseudocyst and therefore a cystojejunostomy was performed.

Patient evolved benignly, without further complications, being discharged on the 10th day after surgical treatment.

### 2.2. Case 2

The second case is of a 59-year-old male with a previous history of alcohol consumption, arterial hypertension, and cholelithiasis. He was treated 2 months previously for an episode of acute pancreatitis that evolved to an asymptomatic pancreatic pseudocyst, smaller than 4 cm in the latest CT.

**Figure 2:**
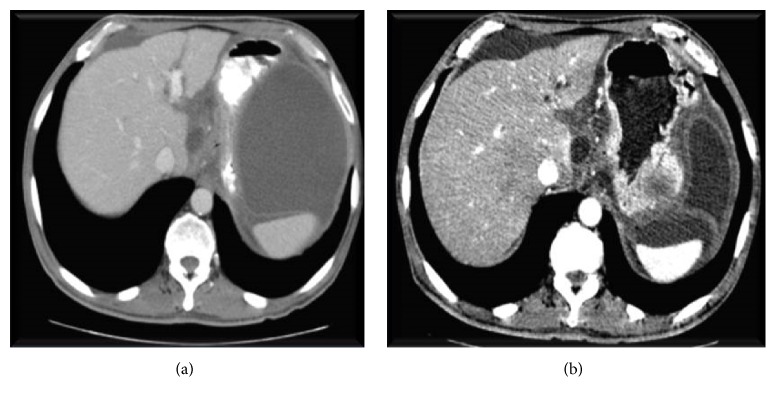
CT scan before and after pseudocyst rupture.

The patient presented to ER with diffuse abdominal pain and food intolerance. The CT scan showed a walled off pancreatic pseudocyst of 11 × 6 cm, with a thick wall (Figure 2(a)). He was also admitted for elective drainage.

At the third day of admission he developed sudden abdominal pain, with an acute abdomen on physical examination. He later became tachycardiac and hypotensive. CT scan showed the presence of free peritoneal fluid, along with reduction in the dimension of the pseudocyst (Figure 2(b)).

He was submitted to emergent laparotomy. Intraoperative findings showed a disseminated chemical peritonitis, with pancreatic ascites. The wall of the pseudocyst was not clearly identifiable. Therefore, the exploration of the pancreatic region revealed intense fibrosis, with aspects of chronic pancreatitis and very difficult exploration of the cephalic region of the pancreas.

Since the cyst walls were not clearly identifiable, it was not possible to perform a drainage of the pseudocyst to the gastrointestinal tract. Therefore, an external drainage and lavage system was established, after extensive peritoneal toilette. After surgery, we introduced total parenteral nutrition, to avoid food intake, and suppressors of pancreatic secretion during 2 weeks. The patient evolved with the onset of pancreatic fistula, controlled by the external drainage.

He was initially treated in the ICU, in the first 5 days, had drainage removed at the 20th day after surgery, and was discharged 30 days postoperatively.

## 3. Discussion

The vast majority of pancreatic pseudocysts with treatment indication are managed electively, by surgical, endoscopic, or percutaneous intervention.

Although emergent presentation is very rare, pancreatic pseudocysts may present as an emergent situation, requiring prompt surgical treatment.

There may be serious complications of pseudocysts if untreated, such as splenic complications, hemorrhage, infection, biliary complications, portal hypertension, and rupture [3].

Pancreatic pseudocyst rupture may occur to the GI tract lumen or to the peritoneal cavity, with the onset of pancreatic ascites, and severe peritonitis.

The ideal surgical option is the drainage of the cyst to the GI tract, through a cystojejunostomy, cystogastrostomy, or cystoduodenostomy. However, when ruptured, the local conditions often make the internal drainage impossible [4]. In those situations, it is imperative to perform a complete peritoneal toilette and a good external drainage.

Regarding the first case, the local conditions were favourable; therefore, we chose to perform a cystojejunostomy, as stated in the literature, allowing for a better and faster recovery.

About the second case, the local conditions were adverse, with intense fibrotic inflammatory process associated with the chronic pancreatitis, and the orifice of the pseudocyst was not clearly identifiable. Towards the impossibility of performing an internal drainage, we chose to do an external drainage with an external lavage system, implying the use of total parenteral nutrition and suppression of pancreatic secretion, as well as longer hospital stay.

Spontaneous pseudocyst rupture of the peritoneum is an extremely rare event, reason why there is few literature reports. The best surgical option is to perform an internal drainage to the GI tract; however, the local conditions may preclude this possibility and obligate doing an external drainage and managing the pancreatic fistula.
